# EUFOREA meeting on defining disease states in allergic rhinitis: towards a unified language in AR

**DOI:** 10.3389/falgy.2024.1531788

**Published:** 2025-02-03

**Authors:** G. K. Scadding, D. M. Conti, S. Scheire, V. Backer, M. Blaiss, L. O. Cardell, W. De Yun, A. K. Ellis, W. Fokkens, A. T. Fox, T. Gilbert Kruz, S. Halken, P. W. Hellings, V. Hox, L. Kalogjera, S. Lau, S. Marinho, M. McDonald, R. Mösges, J. Mullol, S. Nasser, R. Pawankar, D. Price, D. Ryan, G. Scadding, P. Smith, M. Sosa Kostrábová, M. Vazquez-Ortiz, U. Wahn, L. Zhang, P. Gevaert

**Affiliations:** ^1^ Royal National ENT Hospital and University College, London, United Kingdom; ^2^ Escuela de Doctorado UAM, Centro de Estudios de Posgrado, Universidad Autónoma de Madrid, Calle Francisco Tomás y Valiente, no. 2, Ciudad Universitaria de Cantoblanco, Madrid, Spain; ^3^ Allergy and Clinical Immunology Research Unit, Department of Microbiology and Immunology, KU Leuven, Leuven, Belgium; ^4^ Upper Airways Research Laboratory, Department of Head and Skin, Ghent University, Ghent, Belgium; ^5^ Department of Otorhinolaryngology, Head & Neck Surgery, and Audiology, Rigshospitalet, Copenhagen University, Copenhagen, Denmark; ^6^ Medical College of Georgia at Augusta University, Augusta, GA, United States; ^7^ Division of ENT Diseases, Department of Clinical Sciences, Intervention and Technology, Karolinska Institutet, Stockholm, Sweden; ^8^ Department of ENT Diseases, Karolinska University Hospital, Stockholm, Sweden; ^9^ Department of Otolaryngology, Yong Loo Lin School of Medicine, National University of Singapore, Singapore; ^10^ Division of Allergy & Immunology, Department of Medicine, Queen’s University, Kingston, ON, Canada; ^11^ Department of Otorhinolaryngology, Amsterdam University Medical Centres, Amsterdam, Netherlands; ^12^ Guy’s & St Thomas’ Hospitals NHS Foundation Trust, London, United Kingdom; ^13^ Patient Advisory Board, European Forum for Research and Education in Allergy and Airway Diseases, Brussels, Belgium; ^14^ Hans Christian Andersen Children’s Hospital, Odense University Hospital, Odense, Denmark; ^15^ Laboratory of Upper Airways Research, Department of Otorhinolaryngology, University of Ghent, Ghent, Belgium; ^16^ Clinical Department of Otorhinolaryngology, Head and Neck Surgery, University Hospitals Leuven, Leuven, Belgium; ^17^ Department of Otorhinolaryngology, Cliniques Universitaires Saint Luc, Brussels, Belgium; ^18^ Department of Otorhinolaryngology/Head and Neck Surgery, University Hospital Centre “Sestre milosrdnice”, Zagreb School of Medicine, Zagreb, Croatia; ^19^ Department of Pediatric Respiratory Medicine, Immunology and Critical Care Medicine, Charité Universitätsmedizin Berlin, Berlin, Germany; ^20^ Allergy Centre, Wythenshawe Hospital, Manchester University NHS Foundation Trust and The University of Manchester, Manchester, United Kingdom; ^21^ The Allergy Clinic, Johannesburg, South Africa; ^22^ ClinCompetence Cologne GmbH, Cologne, Germany; ^23^ Institute of Medical Statistics and Computational Biology, Faculty of Medicine, University of Cologne, Cologne, Germany; ^24^ Rhinology Unit & Smell Clínic, Department of Otorhinolaryngology, Hospital Clínic Barcelona, Universitat de Barcelona; FRCB-IDIBAPS; CIBERES, Barcelona, Catalonia, Spain; ^25^ Cambridge University Hospitals NHS Foundation Trust, Cambridge, United Kingdom; ^26^ Department of Pediatrics, Nippon Medical School, Bunkyo-ku, Tokyo, Japan; ^27^ Observational and Pragmatic Research Institute, Singapore, Singapore; ^28^ Centre of Academic Primary Care, Division of Applied Health Sciences, University of Aberdeen, Aberdeen, United Kingdom; ^29^ Usher Institute, University of Edinburgh, Edinburgh, Scotland; ^30^ Allergy Department, Royal Brompton Hospital, London, United Kingdom; ^31^ Griffth University, Southport, QLD, Australia; ^32^ Section of Inflammation, Repair and Development, National Heart and Lung Institute, Imperial College London, London, United Kingdom; ^33^ Charite University Medicine, Berlin, Germany; ^34^ Department of Otolaryngology Head and Neck Surgery and Department of Allergy, Beijing TongRen Hospital, Capital Medical University, Beijing, China

**Keywords:** allergic rhinitis, quality of life, definitions, severe allergic rhinoconjunctivitis (SARC), refractory severe allergic rhinoconjunctivitis (R-SARC), biologics, allergen immunotherapy (AIT), control

## Abstract

Allergic rhinitis (AR), the most prevalent immunological disease, affects approximately 400 million individuals globally and can significantly impact quality of life (QoL). Despite nearly 25 years of guidelines, AR remains largely under- diagnosed, suboptimally treated and poorly controlled. In the light of new knowledge and treatment options, there is a necessity to update or revise fundamental AR definitions to facilitate communication across diverse specialties engaged in its treatment and to improve patient care. The European Forum for Research and Education in Allergy and Airway Diseases (EUFOREA) convened a meeting of experts and patient representatives to deliberate the optimal methodology for measuring AR treatment responses and establishing novel treatment goals. This paper presents a consensus on revised AR definitions, including control, severe allergic rhinoconjunctivitis (SARC), refractory severe allergic rhinoconjunctivitis (R-SARC), remission, resolution, improvement, exacerbation, treatable traits (TTs), treat to target, relapse, progression, disease modification, and prevention.

## Introduction

About 30% of the European population is affected by AR (1–3), an inflammatory disorder of the nasal lining that is caused by a reaction to various allergens. AR severity ranges from a minor nuisance to a condition which significantly reduces QoL, work and school attendance and performance (4–7). Furthermore, AR is a risk factor for a number of comorbidities, including allergic asthma (8–10). AR and asthma share a common pathology, based on Th2-inflammation, also relevant to other co-morbid conditions including chronic rhinosinusitis with nasal polyps (CRSwNP).

In light of evolving knowledge and treatment options, there is a growing necessity to (re)define fundamental terms across the diverse specialties engaged in AR management. These should facilitate communication between physicians and patients regarding therapeutic options and treatment goals (11, 12). In the same context, the chronic rhinosinusitis (CRS) expert panel members of the European Forum for Research and Education in Allergy and Airway disease (EUFOREA) has defined disease states in chronic rhinosinusitis, giving rise to the adoption of remission within the treatment goals of CRSwNP (13).

The AR and Its Impact on Asthma (ARIA) initiative is a non-governmental organisation that works in collaboration with the World Health Organization (WHO) to develop guidance on the management of patients with AR. ARIA has provided a useful classification for AR, stressed its frequent links with asthma, evaluated treatments according to Grading of Recommendations-Assessment, Development and Evaluation (GRADE) methodology and initiated mobile technology for disease monitoring (14–17). The most recent iteration of the ARIA document is devoid of a list of definitions, including one pertaining to resolution (15). EUFOREA is an international not-for-profit organisation with a mission of reducing the burden of chronic respiratory diseases by implementing optimal care in daily practice (11, 12). Optimal care implies a correct diagnosis and timely treatment, leading to improvement in QoL of individual patients and cost-savings for society. Following two consensus meetings on asthma in London (April 2023) and CRS in Brussels (June 2023) (13), EUFOREA is now focusing on AR with the objective of reaching a consensus on definitions for disease states and treatment targets. It is our intention that, following this academic exercise, those treating AR will be enabled to communicate more accurately with each other and with patients, allowing identification of areas that require further attention, approaches that warrant further consideration and the strengths and weaknesses of current approaches.

## Methodology

A preliminary virtual meeting was convened to discuss the points and definitions that should be addressed until alignment was reached. The experts were given the opportunity to elaborate on the concepts in advance, thus allowing for a fluid discussion during the in-person debate.

The active participation of internationally renowned specialists in the fields of primary care, ENT, paediatrics, pulmonology, allergology, and immunology facilitated the attainment of an agreement following a full day of discussion in the Royal Society of Medicine of UK in London in June 2024. A patient diagnosed with severe AR participated in the meeting and other patients from the EUFOREA Patient Advisory Board provided feedback on the draft manuscript. A preliminary draft of the consensus document was prepared and subsequently reviewed by a further group of experts, who also served as co-authors. Following the review process, the remaining points of contention were discussed and addressed in virtual web-based meetings until consensus was reached. In 2024, a revised draft was distributed for final review and approval by all contributing authors.

The following topics were included in the discussion: control, severe allergic rhinoconjunctivitis (SARC), refractory severe allergic rhinoconjunctivitis (R-SARC), remission, resolution, improvement, exacerbation, TTs, treat to target, relapse, progression, disease modification, and prevention.

### Unmet needs in AR

Almost a quarter of a century has elapsed since the WHO first established a consensus on AR (14). The creation of the ARIA initiative paved the way for agreement on classifications, unification of diagnostic criteria, and determination of therapeutic measures. Nevertheless, despite the efforts of ARIA and EUFOREA in producing a substantial number of documents, guidelines and educational materials (1–3, 14, 15, 18), the reality is that AR remains a highly prevalent disease and many patients remain uncontrolled (5, 19–21).

Previous research has shown that multiple factors contribute to uncontrolled disease, including disease, patient, environmental, healthcare provider and treatment-related factors which also include the possibility of over-the-counter treatment without assessment or diagnosis of severity (20, 21). A recent observational study by Scheire et al. (5) examined rhinitis control in a real-world setting of Belgian pharmacy patients with persistent rhinitis, with a particular focus on the contribution of patient-related and treatment-related factors. This study revealed that treatment selection was suboptimal, with high usage of systemic corticosteroids and over-the-counter accessibility of nasal decongestants, capable of causing systemic adverse effects and rhinitis medicamentosa, respectively. Additionally, the authors identified that nasal spray technique and adherence to intranasal corticosteroids in real-life is poor with only 10% of patients administering medication correctly (Box 1). A further worry is the purchase of first-generation sedating antihistamines by the public who are unaware of the dangers these represent (22), especially for the treatment of AR symptoms in children in whom they reduce the ability to learn and increase the likelihood of epileptic seizures, among other side effects (23–25).

Box 1 **Errors in nasal spray usage (data extracted from reference 5).**

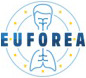
 Forgot to uncap
Did not shake the bottle
Did not clear the nostrils
Pointed toward the nasal septum
Sniffed strongly while/immediately after administering
Held the head backward while administering
Immediately blew the nose after administering

Patients suffering from AR often attempt to self-manage their disease, which can be advantageous from a superficial health economic perspective focused on short-term costs but potentially leads to incorrect treatment choices, inadequate response and increased adverse events (26). However, even among those seeking care from a physician, some patients remain uncontrolled despite optimal combination therapy with both intranasal corticosteroid plus intranasal antihistamine (19). The findings of these studies highlight the unmet need for both enhanced and comprehensive patient education (5, 27–29) and more effective AR treatments and preventive measures (19, 20, 30, 31).

Intranasal glucocorticoids (INCS) represent the preferred treatment option for a significant proportion of individuals with AR. However, there is a tendency for these medications to be employed in a suboptimal manner and subsequently discontinued (1–3, 5, 32). Factors influencing patients' adherence to INCS are illustrated in Table 1. Intentional causes represent the most common category. Disease control is frequently inadequate due to patients' reluctance to adhere to their medication regimens (33, 34). This illustrates the need for patient education as well as devices for administration that improve adherence.

**Table 1:** Adherence to intranasal glucocorticoids (data extracted from reference 5).

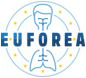	
Intentional non-adherence	Non-intentional non-adherence
No use in case of no symptoms (*n* = 132)	Forgetfulness (*n* = 104)
Only use in case of severe symptoms (*n* = 76)	Ran out of supply (*n* = 36)
Afraid to become dependent (*n* = 34)	Something interfered with daily routine (*n* = 32)
Only a little bit/not effective (*n* = 33)	No time to renew prescription (*n* = 17)
Afraid to use corticosteroids (*n* = 30)	Need for more than advised/prescribed (*n* = 12)
Belief that the effect will be lost upon long-term use (*n* = 22)	
Too many adverse effects (*n* = 8)	
Too expensive for daily use (*n* = 6)	
Other reasons for non-adherence (*n* = 14)	

The EUFOREA AR pocket guidelines (1–3) build on the foundations of ARIA (16) and the British Society for Allergy and Immunology (BSACI) guidelines (18) and present an updated algorithmic approach to the assessment and treatment of AR, at all levels within the health system, with separate diagrams for adults and for children (1–3).

**Figure 1:**
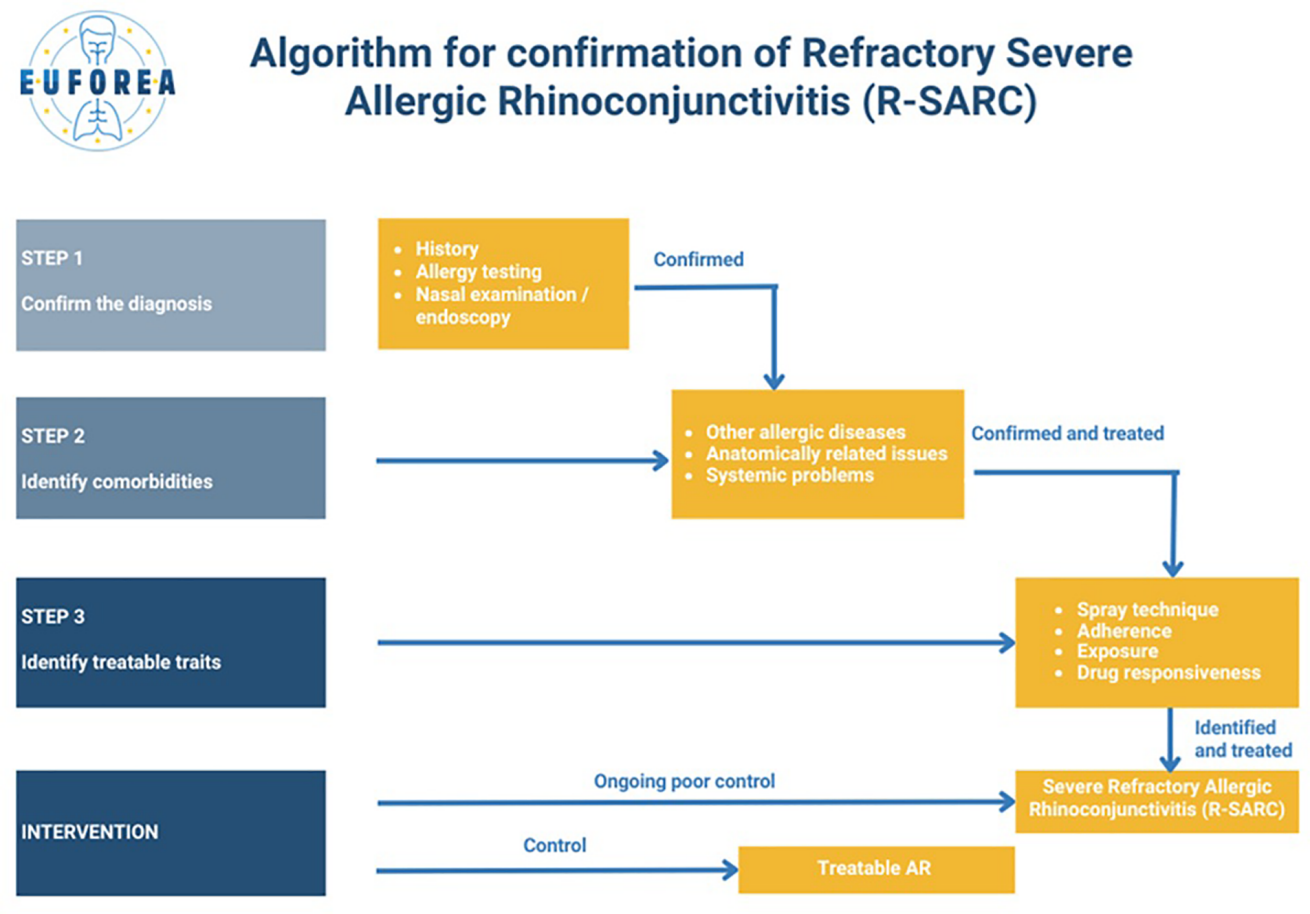
Algorithm for confirmation of refractory severe allergic rhinoconjunctivitis (R-SARC).

Useful definitions of mild, moderate and severe AR have been previously proposed (35–37), together with those for allergic conjunctivitis (38). Although these represent a step forward, the authors believe that they do not address another problematic issue, which is represented by patients with severe disease, with a confirmed diagnosis after re-evaluation, who do not respond to guideline-directed treatments, even after associated comorbidities have been addressed. To address this gap, this group has agreed on two key concepts: a redefinition of severe allergic rhinoconjunctivitis (SARC) and the introduction of a new concept, refractory severe allergic rhinoconjunctivitis (R-SARC).While SARC refers to a condition characterized by allergic inflammation leading to persistent symptoms (>4 days per week for >4 consecutive weeks) which significantly impact QoL, including sleep, work, school or leisure activities, as exemplified by a visual analogue scale (VAS) score ≥70 mm for total nasal symptoms; R-SARC describes patients with ongoing SARC despite optimal guideline-informed pharmacological and non-pharmacological management. This addition is reflected in the algorithm shown in Figure 1.

This milestone opens up a new spectrum of applications, not only in terms of a unified language and consideration of a new level of complexity, but also in terms of the associated therapeutic indications. Specialist treatment reduces AR severity (39).

To date, AIT has proven to be the only treatment capable of modifying disease progression in AR (40–43). Although its inclusion as a therapeutic option is generally accepted and included in guidelines (40–43), there are often barriers to its use, principally due to cost, lack of availability in certain countries, and lack of high-quality vaccine products for some allergens. This leaves a group of patients with severe disease without a therapeutic option. In addition, multiallergic patients are often unsuitable for AIT therapy.

Biologics, such as anti-IgE and anti-cytokine monoclonal antibodies, have recently been explored in the treatment of AR (44). Their potential role is particularly relevant in refractory severe AR with comorbidities (R-SARC) (45), where the use of systemic corticosteroids is frequent and often the only remaining treatment option (2, 3). In many cases of corticosteroid-dependent disease such as asthma or atopic dermatitis, alternatives like biologics have successfully reduced the need for systemic corticosteroids (10). Similarly, better treatment options are needed for this subset of R-SARC patients. The addition of biologics to other therapies has yielded promising outcomes, particularly in patients with severe disease and inadequate control (46, 47). Anti-IgE therapy has been extensively studied in AR; however, two decades ago, its cost-benefit ratio was considered unfavorable, although costs from systemic corticosteroid-related adverse events were ignored (48, 49). With the advent of biosimilars, which should reduce treatment costs, the incorporation of biologics should be considered as a viable option in R-SARC. The combination of AIT and anti-IgE (or other biologics) might allow treatment of patients otherwise deemed too high risk for AIT (typically because of severe/uncontrolled asthma) by reducing the risk of adverse reactions including asthma exacerbations and anaphylaxis. Moreover, the combination would be expected to give more rapid symptom control, giving time for the effects of AIT to be established. The outcomes of AIT are most optimal when used in young children (43). It is the more severely affected child with House dust mite—sensitive AR who is most likely to progress to asthma and other co- morbidities (8), thus such combined therapy in early life has the potential to prevent life-long problems. Research needs to be undertaken in this area. It is our hope that this document will serve as a foundation for the incorporation of biologics into further AR research and subsequently clinical practice.

## Results

The following definitions were discussed and adopted:

### Laying the foundations in AR: control, remission, and resolution

In the context of chronic diseases, including AR, ***control*** is typically the primary objective of treatment (50), given that cure is rarely achievable (50, 51).

**Figure 2:**
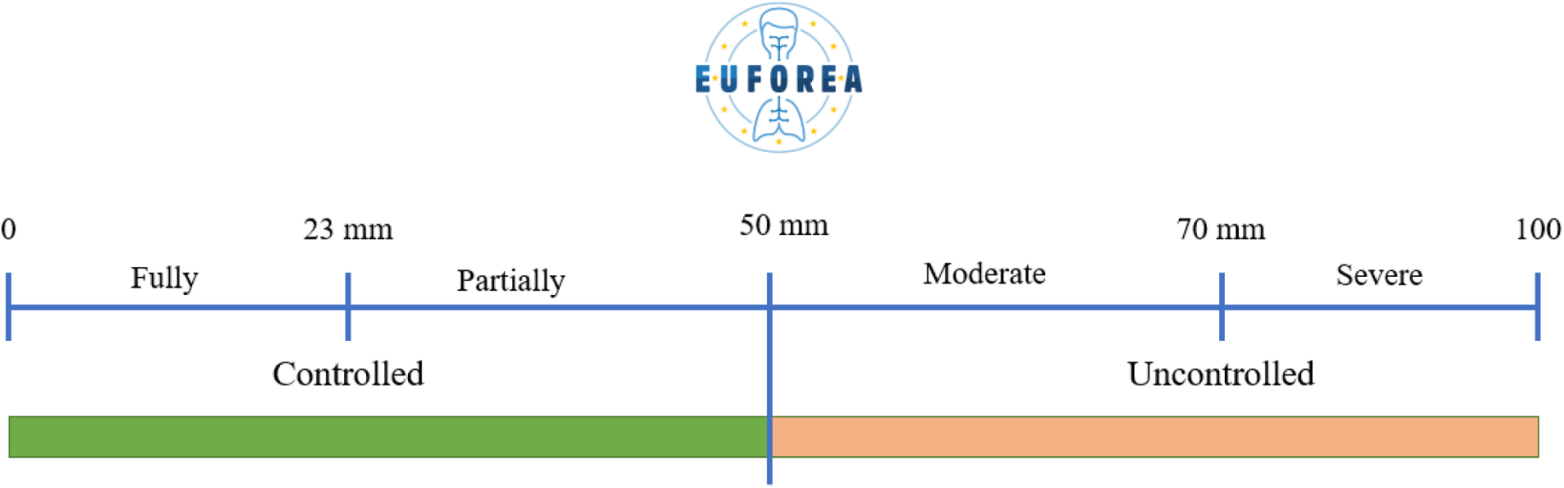
VAS scores and their interpretation.

While the recently introduced categories of uncontrolled disease have been referenced above under the acronyms SARC and R-SARC, this group proposes that ***control*** be defined as “*the extent to which therapy goals are achieved, as determined by the patient and treating physician”*. It is proposed that the VAS be used for this by the patient and the treating physician (48, 49, 52). The proposed limits are as follows: a score of less than or equal to 23 mm (for total symptom severity) would indicate that the patient is well-controlled, while a score greater than 23 mm but less than or equal to 50 mm would indicate that the patient is partially controlled (52, 53) (Figure 2).

In the context of AR, ***clinical remission*** is defined as “*a state or period with low or no disease activity (on or off safe long-term treatment)”*. In a state of remission, patients are free from exacerbations and have a VAS of less than 23 mm (54, 55). The expert board concurred that the definition of ***clinical remission*** should encompass not only patient-reported symptoms and control, but also physician-reported control (54, 55). ***Complete remission*** requires “*normalisation or stabilisation of any underlying pathology in addition to symptomatic remission, as defined for asthma”* occurring off therapy for a period of a year or more (56).

***Resolution*** (or ***cure)*** can be defined as “*a permanent return to a state of normal health, off treatment and without signs of active mucosal disease”* (57, 58). We suggest that ***complete remission*** for a period of 5 years equates to ***resolution***/***cure***.

The objective of treatment should be to achieve ***remission*** in all and ***resolution*** where possible. The available treatments targeting the underlying inflammation of AR and their possible disease-modifying effects must encourage clinicians to aim for more than mere control of the condition in their patients.

### Improvement, exacerbation, and treatable traits

Although ***regression*** is the most commonly used term in the context of other airway diseases, such as CRSwNP (13), this group believes that it carries a negative connotation that is not helpful in the context of AR (59). It is our contention that the term ***improvement*** is the most accurate in this context.

An ***improvement*** of 23 mm in the VAS for total symptoms under therapy indicates effective treatment (60); moreover, an improvement of 30 mm has been consistently associated with improvements in QoL parameters (61). See ***therapeutic response*** below.

An ***exacerbation*** is “*a sudden and pronounced intensification of existing symptoms* (*62*)*, frequently precipitated by an increase in allergen exposure, environmental changes, or concurrent respiratory infections”* (63). Such ***exacerbations*** can have a considerable impact on an individual's daily functioning and QoL, necessitating prompt and effective management (2, 3). It is of the utmost importance to identify and mitigate triggers through environmental controls and patient education in order to prevent ***exacerbations***. In the management of severe exacerbations, pharmacological interventions may be required, including therapeutic escalation (2, 3). An understanding of the underlying mechanisms, including heightened inflammatory responses and airway hyperreactivity, is vital for the development of targeted strategies to predict, prevent and treat exacerbations. In the event of an ***exacerbation*** a step-up approach is typically employed, with rescue treatment often being administered (64). However, the expert board acknowledges the necessity of considering ***TTs*** prior to switching treatments.

TTs represent “*specific phenotypic and endotypic characteristics that can be targeted to optimise patient management*” (65). These traits include the type and severity of allergic response, environmental control, the presence of structural abnormalities (septal deviation, turbinate hypertrophy, or adenoid hypertrophy) or comorbid conditions such as asthma, common cold (including COVID-19)/acute rhinosinusitis, chronic rhinosinusitis with/without nasal polyps or mental health issues, and individual responsiveness to specific treatments. The identification of these traits through comprehensive clinical assessments and biomarker profiling enables a personalised approach to therapy, improving efficacy and reducing adverse effects (2, 3, 65–67). A typical TT fulfils three criteria: identifiable/measurable, clinically relevant, and treatable (68). A ***TTs*** strategy has already been implemented in other airway diseases such as CRSwNP and asthma (68, 69) with improvements in both QoL and response to biologics (68, 69).

Table 2 presents a list of ***TTs*** in patients with AR.

**Table 2:** Treatable traits in AR.

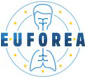
1. Nasal and Sinus-Related Traits
Allergen hypersensitivity with the option of Allergen Immunotherapy (AIT)
Intranasal structural abnormalities
Non-allergic Rhinitis
Nasal hyperreactivity
Chronic rhinosinusitis with and without nasal polyps
Common cold/acute rhinosinusitis (including COVID-19)
Loss of smell [related to severe AR and comorbid nasal diseases (i.e., CRSwNP)]
2. Extranasal Traits
Allergic Conjunctivitis
Cognition and performance issues related to nasal disease
Sleep Disturbance/Quality of sleep and obstructive sleep apnoea
Comorbidities: Asthma, CC/ARS, CRS, otitis media with effusion, food allergy, pollen food syndrome, Obesity, Cystic Fibrosis, Ciliary Dysmotility, mental health problems
3. Behavioral/Risk-Factor Traits
Environmental exposure (allergen and irritant avoidance)
Pets, hobbies, occupational, pollution, indoor/outdoor exposures
Smoking
Occupational allergens
Therapeutic response to treatments

By defining, identifying and addressing these ***TTs***, we aim to improve outcomes of care, including the reduction in oral corticosteroid and/or decongestant use (2, 3) and preventing their adverse effects (39).

### Therapeutic response and treat to target

Therapy in AR is multifaceted, encompassing both pharmacological and non-pharmacological interventions and lifestyle (preventive) measures such as allergen avoidance and nasal douching (70, 71). When preventive measures fail, pharmacotherapy is recommended in addition to continued saline douching. The objective of these interventions is to mitigate symptoms and improve patient QoL, i.e., to achieve a good ***therapeutic response***. First-line treatments typically comprise antihistamines and intranasal corticosteroids, either alone or in fixed (topical) combination, which have been demonstrated to be efficacious in the reduction of nasal congestion, rhinorrhoea and sneezing (70, 71) as well as in olfactory dysfunction which is a marker of severe AR (72) and adults (73).

Immunotherapy, whether subcutaneous or sublingual, represents a disease-modifying approach that provides long-term relief and the potential for remission by inducing immunological tolerance (74–76). New therapies targeting specific immune pathways, such as biologics, show promise in cases where other treatments have failed, although further research is needed to fully elucidate their long-term benefits and safety profiles in AR (48, 49). Optimising therapeutic strategies through personalised medicine, considering patient-specific factors such as allergen sensitivity, comorbid conditions, and genetic predispositions, is pivotal in enhancing treatment outcomes. Several attempts have been made in the past decade within the ARIA and EUFOREA expert panels to define ***therapeutic response***. In most consensus documents (2, 3, 14, 15, 34, 35), a ***therapeutic response*** using the following criteria has been proposed:

- Clinical/symptom score improvement represented by Rhinoconjunctivitis QoL Questionnaire (RQLQ) (77, 78), VAS, and Rhinitis Control Assessment Test (RCAT) (79). The established cut-off variation of 23 mm for VAS was associated with a cut-off variation of 0.5 for RQLQ. Sensitivity analysis with RQLQ and type VI secretion system scales confirmed the aptitude of the cut-off value (23 mm) to discriminate changes in symptoms and QoL (54, 55). A change of 3 points in the RCAT is significant (79).
- Improvement of Sleep quality/tiredness
- Decreased impact on Work/school attendance/performance
- Improvement in recreational activities
- Prevention of progression/improvement of comorbidities
- Reduction of on-demand/as needed pharmacotherapy
- Reduction in ≥1 level of severity (severe -> moderate -> mild) or ≥1 level of control (uncontrolled -> partially controlled -> controlled) could also be considered.
- Upper airway assessment by peak nasal inspiratory flow (PNIF), acoustic rhinometry or rhinomanometry (specialist tools)
- Rhinoscopy (specialist tool)
- A further possible measure is one of olfaction such as Sniffin Sticks (72) since smell loss is proportional to AR severity.
- Further work on such parameters in R-SARC is to be welcomed.

The ***treat to target*** approach to AR represents a paradigm shift towards personalised and goal-directed management (80, 81). This strategy focuses on achieving predefined clinical outcomes such as clinical remission (80, 81). By continuously monitoring patient response, including the use of mobile technology. and adjusting treatment accordingly, clinicians can better address the heterogeneity of patient outcomes in AR (80, 81). Mobile health applications can provide patients with daily management support: medication reminders, pollen count alerts and personalized advice based on symptom tracking. They empower patients to manage their condition more effectively, enhancing adherence and minimizing the impact of AR on daily activities.

Integration of artificial intelligence (AI) in these technologies offers more tailored management. AI can analyze data from a wide array of sources, including environmental monitoring systems and individual health trackers, to predict symptom flare-ups. In addition, AI-driven analytics can contribute to a better understanding of treatment responses and patient behaviours, leading to improved therapeutic strategies. Embracing these technologies in AR management promises to enhance individual patient care and aid in the broader goal of refining treatment protocols and improving outcomes (17, 82–83).

The ***treat to target*** approach often involves the integration of patient-reported outcome measures and objective assessments to precisely tailor interventions. Furthermore, advances in biomarkers and precision medicine are further refining the ***treat to target*** model, enabling more accurate prediction of therapeutic responses and fostering a more dynamic and responsive treatment landscape. The term ***treat to target*** is defined by this group as “*any measure employed in the context of healthcare that is designed to facilitate control, whether that be the control of the patient's symptoms or the control of the pathologic process”*.

### Relapse, progression, and disease modification

A ***relapse*** in AR is “*the recurrence of symptoms following a period of remission”.* This can be triggered by renewed allergen exposure, seasonal changes, or lapses in treatment adherence (84). It is important to gain an understanding of the factors that contribute to relapse in order to develop effective strategies for maintaining long-term control.

***Progression*** in AR involves the worsening of symptoms over time and the potential development of related comorbid conditions such as asthma, acute and chronic rhinosinusitis, and conjunctivitis (84, 85). This ***progression*** is often driven by persistent allergen exposure, ongoing inflammation, and genetic predisposition (84, 85). Early and effective intervention is crucial in altering the disease course and preventing complications. This group proposes to define ***progression*** as a development or worsening of AR symptoms and/or related comorbidities.

The objective of ***disease modification*** in AR is “*to alter the natural course of the disease, thereby achieving sustained symptom relief and improved long-term outcomes”.* This approach is primarily based on AIT (86), which has been demonstrated to induce long-lasting immunological tolerance and to reduce the progression of allergic symptoms, i.e., number of new sensitisations or the development of asthma (87, 88). By targeting the underlying immune mechanisms, such as IgE-mediated hypersensitivity and Th2-driven inflammation, AIT has the potential to significantly diminish the severity and frequency of allergic reactions (86–88). Furthermore, the development of novel biologic therapies that inhibit specific cytokines and immune pathways may represent a promising avenue for disease modification (86–88). Disease-modifying treatments are different from symptomatic treatments as they are able to address the pathogenesis of a disease, preventing progression or leading to a long-term reduction in symptoms even after discontinuation (86–88). ***Disease modification*** may be signalled by ***complete remission***.

This group considers it appropriate to distinguish between the concept of ***disease modification*** in the clinical management of AR to that applied in research. While the former refers to changes induced by treatments or interventions targeting the underlying allergic cause, with effects that persist after treatment cessation the latter needs to be validated using biomarkers, such as IgG_4_.

### Prevention

***Prevention*** in AR involves ’*strategies aimed at reducing the onset and progression of symptoms through a combination of environmental controls, pharmacologic interventions, and lifestyle modifications'*.

There are three types of ***prevention***: primary, secondary, and tertiary prevention that rely on recognition of the aetiology and triggers, early recognition of symptoms and a correct diagnosis/treatment. Primary prevention is intended to prevent disease development, secondary to limit disease progression, and tertiary to reduce associated symptoms and limit sequelae.

***Primary prevention*** focuses on reducing disease incidence by changing causal or predisposing factors, e.g., in the workplace. It encompasses those patients who do not yet have AR but who are at risk of its development (89, 90).

***Secondary prevention*** focuses on early disease detection to return a patient to full health and prevent persistent or extended disease. It refers to patients with early AR or already sensitised and describes measures to be taken to prevent AR from progressing in severity or developing comorbidities by means such as avoidance or reduction of exposure to allergens or irritants or by AIT (8, 43, 90, 91).

***Tertiary prevention*** is defined by a reduction of the impact of ongoing disease and its complications in order to maximize QoL (89). In AR this refers to patients suffering from severe AR and describes measures to be taken to prevent the addition of comorbidities or sequelae (2, 3). This can be achieved by appropriate therapy according to the most recent guidelines (90–92). It is within this context that the incorporation of AIT or biologics into the therapeutic regimen becomes particularly pertinent (90–92).

## Conclusion

This academic exercise sought to initiate improvement in the care and therapeutic management of patients suffering from AR, with a particular focus on those with severe or unresponsive forms of the disease by accurately defining disease states. The result is a list of foundational disease definitions, represented in Table 3.

EUFOREA suggests this glossary may serve as a guide to establish a foundation for improved care for patients with AR.

**Table 3:** Key definitions in AR agreed upon by EUFOREA expert panel members.

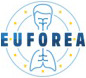	
Severe allergic rhinoconjunctivitis (SARC)	A condition characterized by allergic inflammation leading to persistent symptoms (>4 days per week and >4 consecutive weeks) which significantly impact QoL, including sleep, work, school or leisure activities as exemplified by a VAS score ≥70 mm.
Refractory Severe allergic rhinoconjunctivitis (R-SARC)	Describes those patients with SARC despite adherence to optimal guideline-informed pharmacological and non-pharmacological management.
Control	The degree to which the therapy goals are met, judged by the patient and the treating physician.
Remission	A state or period with low or no disease activity (on or off safe long-term treatment), preferably with a normal nasal examination.
	Complete remission requires normalisation or stabilisation of any underlying pathology in addition to symptomatic remission.
Resolution Cure	A permanent return to the non-disease state
Progression	Development or worsening of the symptoms and/or comorbidities.
Improvement	A clinically significant reduction in symptoms and/or comorbidities.
Regression	Not applicable or useful in the context of AR.
Recurrence	Not applicable or useful in the context of AR.
Exacerbation	A temporary worsening of symptom intensity or severity.
Disease modification in AR	Symptomatic changes caused by treatments or interventions that target the underlying pathophysiology with effects lasting even after treatment cessation
Disease modification in research setting	Disease modification based on symptoms plus validated biomarkers.
Treat to target	The aim to control symptoms to a particular level, often <50 mm on VAS
Relapse	A return to a disease state after a period of remission
Prevention	
Primary	Patients at risk of suffering AR and measures to be taken to prevent it.
Secondary	Patients suffering from early AR or already sensitized and measures to be taken to prevent progression in severity or increased allergen sensitivity or development of co- morbidities such as asthma, often via AIT
Tertiary	Measures to be taken to reduce the impact of ongoing disease and its complications in order to maximize QoL.

## Data Availability

The raw data supporting the conclusions of this article will be made available by the authors, without undue reservation.
